# The *APOA5* rs662799 polymorphism is associated with dyslipidemia and the severity of coronary heart disease in Chinese women

**DOI:** 10.1186/s12944-016-0343-z

**Published:** 2016-09-30

**Authors:** Yanmei Wang, Zhan Lu, Jingxiao Zhang, Yang Yang, Jing Shen, Xiaoming Zhang, Yongyan Song

**Affiliations:** 1 Department of Cardiology, Affiliated Hospital of North Sichuan Medical College, Nanchong, 637000 China; 2 School of Clinical Medicine, North Sichuan Medical College, Nanchong, 637000 China; 3 Department of Nutrition and Health Sciences, University of Nebraska-Lincoln, Lincoln, 68583-0806 NE USA; 4 Department of Radiology, Sichuan Key Laboratory of Medical Imaging, Affiliated Hospital of North Sichuan Medical College, Nanchong, 637000 China; 5 Department of Medical Biochemistry, School of Preclinical Medicine, North Sichuan Medical College, Nanchong, 637000 China

**Keywords:** *APOA5*, rs662799, Coronary heart disease, Lipid, Severity

## Abstract

**Background:**

The *APOA5* rs662799 polymorphism has been widely reported regarding its associations with the plasma lipid levels and the occurrence of coronary heart disease (CHD), whereas its relationship with the severity of CHD has not yet been explored.

**Methods:**

Four hundred and seventy-eight angiografically defined subjects (325 CHD patients and 153 CHD-free controls) were enrolled in this study. The rs662799 polymorphism was genotyped, and the fasting lipid data were collected for all participants. The severity of CHD was evaluated for the CHD patients by using Gensini scores.

**Results:**

The variant C allele of the rs662799 polymorphism was associated with lower levels of HDL-C in CHD-free women, and higher levels of TG and TG/HDL-C in women with CHD (*P* < 0.05 for all). The C allele was associated with higher prevalence of dyslipidemia and higher levels of Gensini scores only in women (*P* < 0.05 for both), but not in men. Multivariate linear regression analysis showed that the rs662799 polymorphism was independently associated with the Gensini scores in women after adjustment for other potential CHD risk factors (Beta = 0.157, 95 % CI: 0.017–0.298, *P* = 0.028).

**Conclusion:**

Our data indicate that the rs662799 polymorphism is associated with dyslipidemia and the severity of CHD in Chinese women.

## Background

Coronary heart disease (CHD) is a major cause of death in developed countries, and in some developing countries like China [1]. CHD is recognized as a multifactorial disease, and dyslipidemia is closely associated with the occurrence and development of CHD. It has been demonstrated that dyslipidemia could account for around 50 % of the population-attributable risk for CHD [2]. Dyslipidemia is the elevation of plasma cholesterol, triglycerides (TG), or both, or a reduction of high-density lipoprotein cholesterol (HDL-C) that contributes to the development of atherosclerosis. Of the dyslipidemia components, low-density lipoprotein cholesterol (LDL-C) can directly deposit on the walls of blood vessels to form atherosclerotic plaques and plasma triglycerides exert their influence on CHD by increasing the coagulation of blood [3]. Plasma cholesterol and TG levels are known to be affected by many factors, among which genetic polymorphisms in the apolipoprotein genes were considered as the key factors [4].

The *APOA1/C3/A4/A5* gene cluster, located on chromosome 11q23, has been shown to be among the most well characterized regions of the human genome with reference to their dynamic associations with plasma lipid levels [5]. *APOA5* is a member of the *APOA1/C3/A4/A5* gene cluster and has been recognized as a key regulator of TG metabolism [6]. This gene is exclusively expressed in liver, and its product, apolipoprotein AV (apoAV), is combined with chylomicron, very low-density lipoprotein (VLDL), high-density lipoprotein (HDL), but not with low-density lipoprotein (LDL). *APOA5*-knockout mice exhibited enhanced TG concentrations and conversely, *APOA5-*overexpressing mice had lower TG levels than those control mice [7]. In humans, a mutation in *APOA5*, which generates a truncated apoAV, has been associated with severe hypertriglyceridemia [8] and hyperchylomicronemia [9]. The underlying mechanisms by which apoAV reduces plasma TG and cholesterol levels are not well-known so far. However, experimental evidence supports the view that this apolipoprotein may function as an activator of lipoprotein lipase in the clearance of TG from the circulation [10]. Furthermore, Vu-Dac et al. [11] reported that *APOA5* is a highly responsive peroxisome proliferator-activated receptor alpha (PPARα)-target gene and fenofibrate, a plasma TG-lowering drug, can increase the *APOA5* expression in hepatocytes by stimulating the PPARα pathway.

Of several variants within *APOA5*, a transition from T to C located upstream of the promoter of this gene results in a rs662799 polymorphism (also known as -1131T>C). The rs662799 polymorphism has been extensively explored regarding its associations with plasma lipid levels and the occurrence of CHD over the past decades. In the literature, some studies demonstrated that this polymorphism is associated with higher levels of TG [12–25], total cholesterol (TC) [17] and LDL-C [18], and lower levels of HDL-C [14–22]. A significant association between this variant and the presence of CHD was also reported in several studies [22–24, 26, 27]. However, whether this polymorphism is associated with the severity of CHD has not been reported before.

Considering the association between the rs662799 polymorphism and dyslipidemia, it is logically plausible to hypothesize a link between the rs662799 polymorphism and the severity of CHD. In this study, a hospital-based study with angiographically defined CHD patients was conducted to systematically investigate the associations of the rs662799 polymorphism with dyslipidemia, the occurrence and severity of CHD. Our study results can provide the opportunity to elucidate the interrelationships among the rs662799 polymorphism, dyslipidemia and CHD.

## Results

### The clinical and genetic characteristics of the study population

As shown in Table 1, the CHD patients had higher age (*P* = 0.001) and higher prevalence of dyslipidemia (*P* = 0.011) and hypertension (*P* = 0.001) than the CHD-free subjects in men; the CHD patients had higher age (*P* < 0.001) and higher prevalence of menopause (*P* = 0.015), dyslipidemia (*P* = 0.027), hypertension (*P* = 0.010) and diabetes (*P* = 0.042) than the CHD-free subjects in women. The CHD patients had higher levels of apoB (*P* = 0.022), TG/HDL-C (*P* = 0.036), TC/HDL-C (*P* = 0.001) and apoB/apoAI (*P* = 0.002), and lower levels of HDL-C (*P* = 0.010) and apoAI (*P* = 0.002) than the CHD-free subjects in men; the CHD patients had higher levels of TG (*P* = 0.016), LDL-C (*P* = 0.017), apoB (*P* = 0.020), TG/HDL-C (*P* = 0.015), TC/HDL-C (*P* = 0.001), LDL-C/HDL-C (*P* < 0.001) and apoB/apoAI (*P* = 0.004) than the CHD-free subjects in women. The *APOA5* rs662799 genotype or allele frequencies did not differ significantly between the CHD patients and the CHD-free subjects in both men and women.

**Table 1:** Clinical and genetic characteristics of the study population

	Men			Women		
	CHD-free (*n* = 81)	CHD (*n* = 205)	*P*	CHD-free (*n* = 72)	CHD (*n* = 120)	*P*
Demographic characteristics						
Age, years	59.88 ± 12.58	64.63 ± 9.51	0.001	59.14 ± 9.69	64.48 ± 8.30	<0.001
Weight, kg	65.35 ± 9.43	65.58 ± 8.54	0.863	59.65 ± 9.39	59.15 ± 9.09	0.902
BMI, kg/m^2^	23.58 ± 3.13	24.04 ± 3.03	0.311	24.26 ± 3.58	24.42 ± 3.68	0.507
Menopause, n (%)	NA	NA		62 (86.11)	115 (95.83)	0.015
Dyslipidemia, n (%)	49 (60.49)	155 (75.61)	0.011	32 (44.44)	73 (60.83)	0.027
Hypertension, n (%)	29 (35.80)	117 (57.07)	0.001	27 (37.50)	68 (56.67)	0.010
Diabetes, n (%)	9 (11.11)	27 (13.17)	0.636	6 (8.33)	23 (19.17)	0.042
Lipid levels						
TG, mmol/L	1.27 ± 0.67	1.58 ± 1.31	0.056	1.42 ± 0.68	1.75 ± 1.10	0.016
TC, mmol/L	3.87 ± 1.01	4.13 ± 1.21	0.087	4.11 ± 1.03	4.40 ± 1.14	0.105
LDL-C, mmol/L	2.54 ± 1.85	2.61 ± 0.95	0.662	2.45 ± 0.77	2.77 ± 0.93	0.017
HDL-C, mmol/L	1.03 ± 0. 29	0.95 ± 0.23	0.010	1.11 ± 0.26	1.06 ± 0.24	0.118
ApoAI, g/L	1.08 ± 0.16	1.01 ± 0.20	0.002	1.13 ± 0.19	1.11 ± 0.18	0.394
ApoB, g/L	0.73 ± 0.24	0.81 ± 0.29	0.022	0.75 ± 0.23	0.84 ± 0.27	0.020
Lp(a), mg/L	264.04 ± 320.60	310.96 ± 332.45	0.140	239.74 ± 249.33	322.45 ± 340.38	0.087
TG/HDL-C	1.41 ± 1.03	1.82 ± 1.55	0.036	1.38 ± 0.83	1.85 ± 1.60	0.015
TC/HDL-C	3.94 ± 1.26	4.52 ± 1.35	0.001	3.75 ± 0.75	4.30 ± 1.31	0.001
LDL-C/HDL-C	2.55 ± 1.51	2.87 ± 1.10	0.052	2.24 ± 0.65	2.73 ± 1.08	<0.001
ApoB/apoAI	0.68 ± 0.25	0.85 ± 0.46	0.002	0.66 ± 0.19	0.78 ± 0.28	0.004
rs662799 Genotype frequency						
TT, n (%)	43 (53.09)	103 (50.24)	0.751	42 (58.33)	63 (52.50)	0.351
TC, n (%)	33 (40.74)	90 (43.90)		28 (38.89)	51 (42.50)	
CC, n (%)	5 (6.17)	12 (5.85)		2 (2.78)	6 (5.00)	
rs662799 Allele frequency						
T allele	0.735	0.722	0.761	0.778	0.738	0.376
C allele	0.265	0.278		0.222	0.263	

*CHD* coronary heart disease, *BMI* body mass index, *NA* not available, *TG* triglycerides, *TC* total cholesterol, *HDL-C* high-density lipoprotein cholesterol, *LDL-C* low-density lipoprotein cholesterol, *apoAI* apolipoprotein AI, *apoB* apolipoprotein B

### Comparisons of the clinical and biochemical variables among the *APOA5* rs662799 genotypes

The demographic variables and lipid levels among the genotypes of the rs662799 polymorphism are shown in Table 2 for the CHD-free subjects and in Table 3 for the CHD patients. HDL-C (*P* = 0.045) levels decreased orderly with the number of C alleles in CHD-free women. TG (*P* = 0.009) and TG/HDL-C (*P* = 0.006) levels increased orderly with the number of C alleles in women with CHD. There were no significant differences in other variables among the genotypes in both men and women.

**Table 2:** Non-lipid variables and lipid levels of the CHD-free subjects by the *APOA5* rs662799 genotypes

	Men				Women			
	TT genotype (*n* = 43)	CT genotype (*n* = 33)	CC genotype (*n* = 5)	*P*	TT genotype (*n* = 42)	CT genotype (*n* = 28)	CC genotype (*n* = 2)	*P*
Non-lipid variables								
Age, years	59.44 ± 12.99	59.88 ± 12.95	63.60 ± 5.89	0.787	59.00 ± 10.86	59.29 ± 7.72	64.50 ± 7.77	0.737
Weight, kg	64.00 ± 8.87	66.53 ± 9.73	69.00 ± 12.08	0.366	59.13 ± 10.76	59.46 ± 8.92	52.50 ± 3.53	0.635
BMI, kg/m^2^	23.30 ± 3.11	23.68 ± 2.96	25.28 ± 4.67	0.483	23.93 ± 4.08	24.48 ± 3.26	20.88 ± 0.85	0.404
Menopause, n (%)	NA	NA	NA		36 (85.71)	24 (85.71)	2 (100.00 %)	0.784
Hypertension, n (%)	14 (32.56)	13 (39.39)	2 (40.00)	0.545	12 (28.57)	14 (50.00)	1 (50.00)	0.079
Diabetes, n (%)	4 (9.30)	5 (15.15)	0 (0.00)	0.898	2 (4.76)	4 (14.29)	0 (0.00)	0.305
Lipid variables								
TG, mmol/L	1.20 ± 0.68	1.24 ± 0.50	2.07 ± 1.06	0.060	1.33 ± 0.65	1.58 ± 0.74	1.15 ± 0.23	0.288
TC, mmol/L	3.91 ± 1.08	3.77 ± 0.93	4.27 ± 0.68	0.554	4.30 ± 1.01	3.89 ± 1.05	3.51 ± 0.09	0.174
LDL-C, mmol/L	2.73 ± 2.44	2.26 ± 0.75	2.73 ± 0.63	0.550	2.59 ± 0.76	2.26 ± 0.77	2.10 ± 0.00	0.161
HDL-C, mmol/L	1.08 ± 0.35	0.98 ± 0.20	0.92 ± 0.25	0.264	1.18 ± 0.25	1.02 ± 0.24	0.92 ± 0.01	0.045
ApoAI, g/L	1.10 ± 0.15	1.06 ± 0.17	1.08 ± 0.17	0.705	1.16 ± 0.16	1.09 ± 0.22	0.99 ± 0.16	0.152
ApoB, g/L	0.71 ± 0.25	0.73 ± 0.24	0.85 ± 0.22	0.456	0.77 ± 0.25	0.73 ± 0.21	0.66 ± 0.10	0.680
Lp(a), mg/L	251.09 ± 323.39	280.68 ± 327.62	272.26 ± 321.52	0.926	236.26 ± 248.07	260.84 ± 271.67	211.95 ± 17.32	0.911
TG/HDL-C	1.29 ± 0.97	1.38 ± 0.78	2.62 ± 2.11	0.066	1.22 ± 0.78	1.63 ± 0.87	1.25 ± 0.25	0.131
TC/HDL-C	3.86 ± 1.43	3.91 ± 0.96	4.85 ± 1.24	0.247	3.69 ± 0.73	3.85 ± 0.82	3.83 ± 0.07	0.693
LDL-C/HDL-C	2.64 ± 1.92	2.36 ± 0.81	3.11 ± 0.92	0.506	2.23 ± 0.63	2.24 ± 0.69	2.29 ± 0.02	0.992
ApoB/apoAI	0.66 ± 0.27	0.69 ± 0.22	0.81 ± 0.22	0.424	0.66 ± 0.19	0.67 ± 0.17	0.69 ± 0.21	0.951

*CHD* coronary heart disease, *BMI* body mass index, *NA* not available, *TG* triglycerides, *TC* total cholesterol, *HDL-C* high-density lipoprotein cholesterol, *LDL-C* low-density lipoprotein cholesterol, *apoAI* apolipoprotein AI, *apoB* apolipoprotein B

**Table 3:** Non-lipid variables and lipid levels of the CHD patients by the *APOA5* rs662799 genotypes

	Men				Women			
	TT genotype (*n* = 103)	CT genotype (*n* = 90)	CC genotype (*n* = 12)	*P*	TT genotype (*n* = 63)	CT genotype (*n* = 51)	CC genotype (*n* = 6)	*P*
Non-lipid variables								
Age, years	64.97 ± 9.34	64.17 ± 10.00	65.17 ± 7.60	0.827	65.17 ± 7.49	63.82 ± 9.20	62.40 ± 9.29	0.589
Weight, kg	65.71 ± 8.27	65.57 ± 9.13	64.00 ± 7.05	0.822	59.07 ± 8.14	58.59 ± 10.35	66.17 ± 5.63	0.156
BMI, kg/m^2^	24.17 ± 3.02	23.86 ± 3.10	23.81 ± 2.74	0.762	24.15 ± 3.06	24.55 ± 4.48	26.59 ± 1.83	0.298
Menopause, n (%)	NA	NA	NA		62 (98.41)	48 (94.12)	5 (83.33)	0.068
Hypertension, n (%)	57 (55.34)	55 (61.11)	5 (41.47)	0.988	42 (66.7)	23 (45.10)	3 (50.00)	0.065
Diabetes, n (%)	16 (15.53)	10 (11.11)	1 (8.33)	0.303	13 (20.6)	9 (17.65)	1 (16.67)	0.674
Lipid variables								
TG, mmol/L	1.36 ± 0.74	1.76 ± 1.73	1.83 ± 0.91	0.073	1.51 ± 0.86	1.96 ± 1.27	3.01 ± 1.55	0.009
TC, mmol/L	4.08 ± 1.18	4.24 ± 1.29	3.82 ± 0.84	0.448	4.31 ± 1.23	4.48 ± 1.03	4.44 ± 1.22	0.722
LDL-C, mmol/L	2.57 ± 0.91	2.71 ± 1.03	2.30 ± 0.61	0.307	2.70 ± 1.03	2.82 ± 0.79	2.94 ± 1.04	0.749
HDL-C, mmol/L	0.96 ± 0.20	0.95 ± 0.23	0.82 ± 0.24	0.124	1.09 ± 0.24	1.03 ± 0.24	0.91 ± 0.24	0.149
ApoAI, g/L	1.00 ± 0.16	1.02 ± 0.20	1.00 ± 0.15	0.861	1.13 ± 0.16	1.08 ± 0.20	1.11 ± 0.18	0.234
ApoB, g/L	0.80 ± 0.30	0.83 ± 0.30	0.77 ± 0.19	0.618	0.80 ± 0.27	0.88 ± 0.26	0.83 ± 0.33	0.255
Lp(a), mg/L	328.47 ± 362.37	271.98 ± 285.15	412.95 ± 371.18	0.271	369.27 ± 374.08	258.82 ± 274.92	441.90 ± 437.05	0.164
TG/HDL-C	1.53 ± 1.04	1.98 ± 1.81	2.60 ± 1.78	0.057	1.52 ± 1.00	2.14 ± 2.02	3.83 ± 2.73	0.006
TC/HDL-C	4.40 ± 1.41	4.59 ± 1.30	4.86 ± 1.17	0.411	4.07 ± 1.27	4.53 ± 1.33	4.99 ± 1.19	0.080
LDL-C/HDL-C	2.80 ± 1.16	2.95 ± 1.09	2.90 ± 0.68	0.665	2.57 ± 1.08	2.88 ± 1.07	3.26 ± 0.86	0.150
ApoB/apoAI	0.85 ± 0.45	0.87 ± 0.51	0.78 ± 0.19	0.808	0.72 ± 0.26	0.84 ± 0.29	0.86 ± 0.29	0.057

*CHD* coronary heart disease, *BMI* body mass index, *NA* not available, *TG* triglycerides, *TC* total cholesterol, *HDL-C* high-density lipoprotein cholesterol, *LDL-C* low-density lipoprotein cholesterol, *apoAI* apolipoprotein AI, *apoB* apolipoprotein B

### The frequencies of the *APOA5* rs662799 genotypes and alleles in the non-dyslipidemic and dyslipdemic subjects

The distribution of the rs662799 genotypes and alleles was significantly different between the non-dyslipidemic subjects and dyslipidemic subjects in women (Table 4). The frequencies of TC (*P* = 0.022) and CC (*P* = 0.050) genotypes were higher in the dyslipidemic women than in the non-dyslipidemic women. The C allele frequency was higher in the dyslipidemic women than in the non-dyslipidemic women (*P* < 0.001). There were no significant differences in the distribution of genotypes or alleles between the non-dyslipidemic subjects and the dyslipidemic subjects in men.

**Table 4:** The distribution of the *APOA5* rs662799 genotypes and alleles in the non-dyslipidemic and dyslipdemic subjects

	Men			Women		
	Non-dyslipidemic (*n* = 82)	Dyslipidemic (*n* = 204)	*P*	Non-dyslipidemic (*n* = 87)	Dyslipidemic (*n* = 105)	*P*
Genotype frequency						
TT, n (%)	45 (54.88)	101 (49.51)	0.280	59 (67.82)	46 (43.81)	<0.001
TC, n (%)	34 (41.46)	89 (43.63)		28 (32.18)	51 (48.57)	
CC, n (%)	3 (3.66)	14 (6.86)		0 (0.00)	8 (7.62)	
Allele frequency						
T allele	0.756	0.713	0.299	0.839	0.681	<0.001
C allele	0.244	0.287		0.161	0.319	

### The Gensini score levels of the CHD patients according to the *APOA5* rs662799 genotypes

**Fig. 1:**
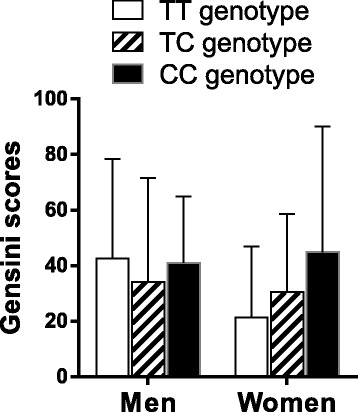
The Gensini score levels of the CHD patients according to the *APOA5* rs662799 genotypes. The Gensini score levels were significantly different among the women with different rs662799 genotypes (*P* < 0.05)

As shown in Fig. 1, the Gensini score levels increased orderly with the number of C alleles in women patients (*P* = 0.045). In men patients, the Gensini score levels were similar among the genotypes.

### Linear regression analysis of the *APOA5* rs662799 polymorphism and other variables with the Gensini scores in the CHD patients

The results of the linear regression analysis of the potential CHD risk factors and the Gensini scores are shown in Table 5. In men, univariate linear regression analyses were conducted and 9 viaribles [hypertension, TC, LDL-C, apoAI, apoB, Lp(a), TC/HDL-C, LDL-C/HDL-C and apoB/apoAI] were found to be associated with the Gensini scores (*P* < 0.1 for all). Then the 9 variables were taken into the stepwise multivariate linear regression analysis and Lp(a) (*P* = 0.035) and apoB/apoAI (*P* < 0.001) were found to be independently associated with the Gensini scores after controlling for the confounding variables including hypertension, TC, LDL-C, apoAI, apoB, TC/HDL-C and LDL-C/HDL-C. In women, 9 viaribles [*APOA5* rs662799 polymorphism, TC, LDL-C, apoAI, apoB, Lp(a), TC/HDL-C, LDL-C/HDL-C and apoB/apoAI] were found to be associated with the Gensini scores in univariate linear regression analyses (*P* < 0.1 for all). Then the 9 variables were taken into the stepwise multivariate linear regression analysis and *APOA5* rs662799 polymorphism (*P* = 0.028), Lp(a) (*P* = 0.009) and apoB/apoAI (*P* = 0.005) were found to be independently associated with the Gensini scores after controlling for the confounding variables including hypertension, TC, LDL-C, apoAI, apoB, TC/HDL-C and LDL-C/HDL-C.

**Table 5:** Linear regression analysis of the Gensini scores (dependent variable) and the potential CHD risk factors (independent variables) in CHD patients

Variables	Men				Women			
	Univariate linear regression	*P*	Multivariate linear regression	*P*	Univariate linear regression	*P*	Multivariate linear regression	*P*
	Beta (95 % CI)		Beta (95 % CI)		Beta (95 % CI)		Beta (95 % CI)	
*APOA5* rs662799	−0.053 (−0.164–0.057)	0.342			0.187 (0.041–0.334)	0.013	0.157 (0.017–0.298)	0.028
Age	0.001 (−0.006–0.008)	0.777			0.008 (−0.003–0.018)	0.157		
Weight	0.003 (−0.005–0.011)	0.530			−0.004 (−0.015–0.006)	0.391		
BMI	0.018 (−0.004–0.041)	0.113			−0.007 (−0.003–0.019)	0.591		
Menopause	NA	NA			−0.031 (−0.474–0.411)	0.889		
Hypertension	0.063 (0.013–0.112)	0.013			0.001 (−0.065–0.066)	0.981		
Diabetes	0.106 (−0.091–0.303)	0.289			0.021 (−0.205–0.247)	0.854		
TG	−0.016 (−0.067–0.036)	0.556			0.031 (−0.047–0.109)	0.427		
TC	0.059 (0.004–0.113)	0.035			0.074 (−0.003–0.151)	0.061		
LDL-C	0.096 (0.027–0.165)	0.007			0.095 (0.001–0.189)	0.048		
HDL-C	−0.113 (−0.401–0.176)	0.442			−0.221 (−0.586–0.143)	0.232		
ApoAI	−0.384 (−0.717– −0.051)	0.024			−0.841 (−1.330– −0.352)	0.001		
ApoB	0.372 (0.147–0.597)	0.001			0.430 (0.105–0.755)	0.010		
Lp(a)	0.192 (0.038–0.346)	0.015	0.162 (0.012–0.312)	0.035	0.360 (0.152–0.568)	0.001	0.278 (0.071–0.486)	0.009
TG/HDL-C	−0.001 (−0.046–0.043)	0.957			0.012 (−0.041–0.065)	0.647		
TC/HDL-C	0.073 (0.025–0.121)	0.003			0.079 (0.012–0.146)	0.021		
LDL-C/HDL-C	0.101 (0.042–0.160)	0.001			0.094 (0.013–0.175)	0.024		
ApoB/apoAI	0.277 (0.137–0.416)	<0.001	0.261 (0.124–0.398)	<0.001	0.628 (0.332–0.923)	<0.001	0.446 (0.137–0.756)	0.005

*CHD* coronary heart disease, *BMI* body mass index, *NA* not available, *95 % CI* 95 % confidence interval, *TG* triglycerides, *TC* total cholesterol, *ApoAI* apolipoprotein AI, *ApoB* apolipoprotein B, *HDL-C* high-density lipoprotein cholesterol, *LDL-C* low-density lipoprotein cholesterol, *Lp(a)* lipoprotein (a)

## Discussion

The present study demonstrated that the rs662799 polymorphism was independently associated with the severity of CHD in Chinese women patients. To our knowledge, this is the first demonstration of a significant association between the rs662799 polymorphism and the severity of CHD, although the associations of the variant with abnormal lipid profiles and the occurrence of CHD have been reported in Chinese [22, 26, 27], Hungarian [23] and Japanese [24].

The significant association between the rs662799 polymorphism and the severity of CHD was only observed in women patients, but not in men patients. The gender-specific effects of the rs662799 polymorphism on the severity of CHD are not well understood. One reason could be that the rs662799 polymorphism was associated with dyslipidemia only in women (Table 4). The women carrying one or more C alleles of the rs662799 polymorphism had higher prevalence of dyslipidemia than those without this allele. It is logical to suppose that the C allele carrying women experienced faster deposition of lipids in the walls of coronary arteries and had severer coronary stenosis than those without this allele. Another raised question could be that why the rs662799 polymorphism was associated with higher prevalence of dyslipidemia only in women, but not in men in our study population. This can be partly explained by the different lifestyles between Chinese men and women. It has been reported that the rs662799 polymorphism had interaction with lifestyles on the lipid levels in Asians [28]. The gender-specific effects of the rs662799 polymorphism on lipid levels were also reported in other populations. In Turkish adults, Komurcu-Bayrak et al. [14]. demonstrated that the C carriers had higher fasting TG levels in both genders, but lower HDL-C levels only in women than the non-carriers. In our study population, the CHD-free subjects were categorized into three subgroups [subjects with normal coronary arteries, subjects with coronary atherosclerosis and subjects with minimal coronary stenosis (stenosis < 50 %)] according to the extent of coronary atherosclerosis. Interestingly, there was a weak trend in the association between the rs662799 polymorphism and the extent of coronary atherosclerosis in CHD-free women, but not in men (data not shown).

Our data did not support a significant association between the rs662799 polymorphism and the occurrence of CHD, although an independent and significant association was observed between the rs662799 polymorphism and the severity of CHD in women. This result is beyond our understanding since a series of case–control studies demonstrated a significant association between the rs662799 polymorphism and the occurrence of CHD [22–24, 26, 27]. One of the reasons could be that the control group in our study was not made up of healthy individuals, but the subjects who underwent angiography for suspected CHD. In the control group, 54.90 % of them had normal coronary arteries, 33.99 % of them had coronary atherosclerosis and 11.11 % of them had minimal coronary stenosis (stenosis < 50 %). Some other studies [21, 29] which demonstrated negative association between the rs662799 polymorphism and the occurrence of CHD also enrolled the subjects with coronary atherosclerosis or minimal coronary stenosis as controls. On the other hand, all the studies [22–24, 26, 27] which concluded a positive association between the rs662799 polymorphism and the occurrence of CHD used healthy individuals as controls.

The C allele frequency of the rs662799 polymorphism was 0.26 in the present study population, which is consistent with the ranges reported for other Asians, including Chinese (0.29–0.39) [16, 17, 22, 26], Korean (0.31) [15] and Japanese (0.33–0.35) [24], but is higher than those reported for Caucasians (0.06–0.11) [13, 20, 21, 23]. The rs662799 polymorphism might have some effects on the expression pattern of *APOA5*. The circulating levels of apoAV protein were affected by the rs662799 polymorphism in several studies, but the results were inconsistent [30, 31]. Kang et al. [30] demonstrated that the C allele carriers had higher levels of apoAV and TG than the non-carriers in type 2 diabetes patients, whereas Jang et al. [31] reported that the C allele carriers had lower levels of apoAV but higher levels of TG in healthy subjects and CHD patients. ApoAV is combined with chylomicron, VLDL, HDL and considered as a key regulator of TG metabolism. The plasma levels of apoAV were reported to be negatively associated with the TG levels [7–9]. Therefore, the association between the rs662799 polymorphism and the severity of CHD could be mediated by apoAV and hypertriglyceridemia.

Our findings should be considered in the context of several potential limitations. Firstly, the small sample size in each group may have limited the power to detect a significant relationship. Secondly, the control group was not made up of healthy individuals, but rather the subjects who underwent angiography with suspected CHD at our hospital, which may have led to a selection bias. However, it was difficult to enroll healthy subjects who underwent coronary angiography from general population. Thirdly, the patients included in this study were exclusively Chinese Han people, and therefore our findings may not apply to other ethnic origins.

## Conclusions

The rs662799 polymorphism is significantly associated with dyslipidemia and the severity of CHD in Chinese women, but further investigations with large sample size and multi-ethnicities are required to validate the findings from the present study.

## Methods

### Study participants

This study was designed as a hospital-based study. A total of 478 consecutive and unrelated adult subjects who underwent coronary angiography for suspected CHD at the Department of Cardiology, the Affiliated Hospital of North Sichuan Medical College (Nanchong, China) were enrolled in the study between April 2014 and July 2015. Of these subjects, 325 subjects were diagnosed with CHD, while the rest 153 subjects were free of CHD and considered as the control group. Subjects taking lipid lowering drugs or the drugs that might affect the glucose or lipid metabolism were excluded from the study. In order to enlarge the sample size, those who took the drugs which were thought not to affect plasma lipid levels were still enrolled in this study. Subjects with renal or hepatic dysfunction, active inflammatory disease, significant valvular disease, myocarditis, and malignant disease were also excluded from the study. All the subjects were Chinese Han people. The Han people are the largest ethnic group in East Asia, constituting approximately 92 % of the population of Mainland China, 98 % of the population of Taiwan, and 74 % of the population of Singapore. The tenets of the Declaration of Helsinki were adhered to in all the procedures reported in the article. The study protocol was reviewed and approved by the Ethics Committee of the Affiliated Hospital of North Sichuan Medical College. Signed informed consent was provided by all the participants or their guardians prior to their participation in the study.

### Definitions

According to the Third Report of the National Cholesterol Education Program (NCEP) Expert Panel on Detection, Evaluation, and Treatment of High Blood Cholesterol in Adults (ATP III), dyslipidemia was defined as the presence of one or more of the following conditions: TG ≥ 2.26 mmol/L, TC ≥ 6.22 mmol/L, LDL-C ≥ 4.14 mmol/L and HDL-C < 1.04 mmol/L [32]. Smoking was defined as regular cigarette smoking. Hypertension was defined as the measurement of systolic/diastolic blood pressure higher than 140/90 mmHg or active use of antihypertensive drugs. Diabetes mellitus was defined as the fasting glucose levels above 126 mg/dL or active use of antidiabetic drugs or insulin. Body mass index (BMI) was calculated by dividing weight by height squared (kg/m2).

### Biochemical measurement

Fasting blood samples were taken on the first morning of the in-hospital day when no lipid-lowering drugs were used. Samples were immediately shipped to the Department of Clinical Laboratory of the Affiliated Hospital of North Sichuan Medical College for measurement of plasma lipids. TG, TC, LDL-C and HDL-C were measured directly by enzymatic methods. ApoB, apoAI and Lp(a) were measured by immunoturbidimetric assays. All the measurements were carried out using an automatic clinical chemistry analyzer (Beckman Coulter AU5800, USA). The lipoprotein ratios were also calculated.

### Diagnosis of CHD and evaluation of the severity of CHD

CHD was diagnosed in the patients who had angiographic evidence of stenosis greater than 50 % in at least one major coronary artery. Those with normal coronary arteries, coronary atherosclerosis, or minimal stenosis (less than 50 %) in any of the major coronary arteries were considered as CHD-free control subjects. Two experienced cardiologists who were unaware of the clinical history and laboratory results of the subjects performed the coronary angiography. Standard coronary angiography with at least four views of the left coronary system and two views of the right coronary artery was performed using the Judkins technique by Allura Xper FD20 (Philips Medical Systems Nederland B.V. Netherlands). The Gensini scoring system [33] was used to assess the severity of CHD. In this system, the lumen narrowing of coronary arteries is graded as 1 point for 1–25 % narrowing, 2 points for 26–50 % narrowing, 4 points for 51–75 % narrowing, 8 points for 76–90 % narrowing, 16 points for 91–99 % narrowing, and 32 points for complete occlusion. Each score is then multiplied by a factor that takes into account the importance of the lesion’s position in the coronary arterial tree, for example, 5 points for the left main coronary artery, 2.5 points for the proximal left anterior descending branch (LAD) or proximal left circumflex branch (LCX), 1.5 points for the middle LAD or middle LCX, and 1 point for the distal LAD, distal LCX or right coronary arteries. The Gensini score was calculated as the sum of the scores for all coronary arteries.

### Statistical analysis

Continuous data were presented as mean ± standard deviation (SD) unless otherwise stated. Continuous variables were tested for normality, and log transformation was conducted for those which did not conform to a normal distribution. The differences between the CHD patients and the CHD-free subjects, the dyslipidemic subjects and the non-dyslipidemic subjects, or among the subjects with different genotypes were calculated by Chi-square test for categorical variables, and one-way ANOVA analysis for continuous variables. The Gensini score levels among the patients with different genotypes were compared by one-way ANOVA analysis. The P values were adjusted by using the Bonferroni correction in multiple comparisons of the variables among the rs662799 genotypes. The associations between the rs662799 polymorphism and the other potential CHD risk factors and the Gensini scores were analyzed by univariate and stepwise multivariate linear regression analyses; the variables with a *P* value less than 0.1 in univariate analysis were taken into the multivariate analysis. The results of regression analysis were presented as Beta with 95 % confidence interval (95 % CI). All *P* values were two-tailed and the differences were considered as significant if *P* ≤ 0.05. All statistical analyses were done by using 13.0 version SPSS (Chicago, IL, USA).

## Abbreviations

ApoAI: Apolipoprotein AI
ApoB100: Apolipoprotein B100
BMI: Body mass index
CHD: Coronary heart disease
HDL-C: High-density lipoprotein cholesterol
LDL-C: Low-density lipoprotein cholesterol
Lp(a): Lipoprotein (a)
TC: Total cholesterol
TG: Triglycerides
